# Multiple Substance Use Disorders and Self-Reported Cognitive Function in U.S. Adults: Associations and Sex-Differences in a Nationally Representative Sample

**DOI:** 10.3389/fpsyt.2021.797578

**Published:** 2022-01-11

**Authors:** Zoe Bourgault, Dafna Sara Rubin-Kahana, Ahmed Nabeel Hassan, Marcos Sanches, Bernard Le Foll

**Affiliations:** ^1^Translational Addiction Research Laboratory, Centre for Addiction and Mental Health, Toronto, ON, Canada; ^2^Department of Pharmacology and Toxicology, University of Toronto, Toronto, ON, Canada; ^3^Department of Psychiatry, Faculty of Medicine, University of Toronto, Toronto, ON, Canada; ^4^Child and Youth Mental Health Division, Centre for Addiction and Mental Health, Toronto, ON, Canada; ^5^Campbell Family Mental Health Research Institute, Centre for Addiction and Mental Health, Toronto, ON, Canada; ^6^Department of Psychiatry, King Abdul-Aziz University, Jeddah, Saudi Arabia; ^7^Biostatistics Core, Centre for Addiction and Mental Health, Toronto, ON, Canada; ^8^Departments of Family and Community Medicine, University of Toronto, Toronto, ON, Canada; ^9^Institute of Medical Science, University of Toronto, Toronto, ON, Canada

**Keywords:** polysubstance use, substance use disorder, cognition, sex differences, addiction

## Abstract

Polysubstance use is a growing public health concern that has been associated with poor clinical outcomes. Compared to single-drug users, this population suffers greater deficits in cognitive function, which hinder treatment success and recovery. Despite its high prevalence and poor prognosis, epidemiological research on polysubstance use and accompanying cognitive profile is lacking. We investigated associations between numbers of past-year co-occurring substance use disorders (SUDs) and self-reported cognitive function using data from the National Epidemiologic Survey for Alcohol and Related Conditions III (NESARC-III). Regression analyses revealed a significant negative association between cognitive scores and numbers of past-year SUDs, which was moderated by sex. After adjusting for confounding variables, greater numbers of SUDs were associated with declining self-reported cognitive function, and this relationship was stronger among females. Our findings expand on current literature on cognitive impairments among polysubstance users and provide a novel, nuanced description of this relationship among the general population. We highlight the need for targeted and individualized treatment approaches in order to improve outcomes in this population.

## Introduction

The majority of individuals affected by substance use disorders (SUDs) report the habitual use of multiple drugs (1). In clinical settings, it is common for SUD patients to have a history of polysubstance use or to meet diagnostic criteria for multiple SUDs at a time (1–3). Polysubstance users represent up to 91% of treatment-seeking drug-users, who consume an average of 3.5 concurrent substances (4). These individuals tend to be males, young adults, and from lower socioeconomic status (5). Compared to single-drug users, polysubstance users are younger at the onset of drug use, and suffer a more persistent disorder course, complicated by higher rates of comorbid psychiatric diagnoses and medical problems (2, 3, 6). Overall, both epidemiological and prospective studies have established polysubstance use as a growing public health concern associated with poorer outcomes and higher rates of mortality (1, 2, 7, 8).

SUDs are commonly associated with deficits in cognitive function. A large body of neuropsychological research has described significant impairments in executive function, attention, and decision-making that are thought to contribute to SUD onset and relapse (9, 10). Cognitive dysfunction has been associated with predictors of treatment failure such as reduced attendance, shorter periods of drug abstinence, and higher rates of dropout (11–13). There is substantial evidence indicating that polysubstance use results in more severe neurocognitive deficits than single-drug use (14–16). This has been demonstrated as poorer performance in tasks assessing processes such as executive function, processing speed, working memory, and visuospatial ability (14, 17, 18). For instance, when compared to patients with alcohol use disorder, polysubstance users show lower performance on multiple measures of learning and memory, and score higher on measures of impulsivity (16). Neuroimaging studies have associated these deficits with structural and chemical abnormalities that differ from those seen in both single-drug users and controls (18–20). Whether impairments are a consequence of the repeated use of multiple drugs, or alternatively predispose certain individuals to developing multiple SUDs is unclear.

In addition to greater cognitive deficit, polysubstance use has been associated with significantly reduced cognitive recovery during drug abstinence. Improvements in cognitive performance have been correlated to duration of abstinence in single drug-users, whereas impaired performance persists over time in those who used multiple substances (14). Even following 1 year of abstinence, polysubstance users still showed lower cognitive functions compared to controls (21). The severe and enduring neuropsychological consequences associated with the abuse of multiple drugs may thus explain the poorer outcomes in this population.

A number of studies have described neurocognitive deficits that occur with polysubstance use. However, research distinguishing between polysubstance use of different numbers of drugs is lacking. One study found evidence of an association between declining performance on a verbal memory task and number of DSM-IV diagnoses of substance dependence in women (22). Neuroimaging research has also found structural abnormalities in polysubstance users that increase in severity with greater numbers of substances used. The prefrontal localization of these abnormalities may reflect the cognitive deficits seen in this population (23, 24). In the present study, our primary aim was to determine whether a relationship exists between the number of past-year co-occurring SUDs and self-reported cognitive function. We also sought to investigate whether the strength of this relationship is moderated by sex, due to the significant sex-differences in the clinical presentation of SUDs. Compared to males, females suffering from SUDs are more likely to meet diagnostic criteria for comorbid psychiatric disorders, report higher levels of distress, and are more likely to have a history of trauma or abuse (25). Females have also been found to progress more quickly from initial drug exposure to dependence (26). Studies on adolescent substance use have found that females appear to be more severely affected by cognitive deficits compared to males (27, 28). Current literature has not adequately addressed sex differences in the clinical presentation of polysubstance use. Importantly, potential sex-specific neurocognitive outcomes may have significant treatment implications and require further study.

We addressed these questions using data from the National Epidemiologic Survey on Alcohol and Related Conditions-III (NESARC-III). As the majority of polysubstance users do not seek treatment (6), this large national sample provides a more adequate representation than would a clinical sample. We hypothesized that self-reported cognition would worsen with increasing numbers of past-year SUDs. Regarding sex differences, we hypothesized that the relationship between self-reported cognitive function and number of past-year SUDs would be stronger among females. To the best of our knowledge, this is the first study assessing the association between cognitive function and numbers of SUDs in a national survey. We are also the first to investigate the effect of sex on this relationship.

## Methods

### Sample

The NESARC-III is a cross-sectional, nationally representative survey conducted in the United States in 2012-2013. The participants were 36,309 civilian adults in households and selected group quarters (29). Our study included 35,916 respondents after excluding 393 respondents (1.1% of the sample) who had missing responses for any of the Executive Function Index (EFI) items.

The survey respondents were selected through a multi-stage probability sampling procedure, and data were weighted to represent the US population based on the 2012 American Community Survey (“Income and Poverty in the United States: 2016”). Face-to-face interviews took place in respondents' homes. The household response rate was 72%, the person-level response rate was 84%, and the overall response rate was 60.1%. NESARC-III methodology is described further elsewhere (29). The National Institutes of Health institutional review boards approved the survey.

### Measures

#### Executive Function Index Scales

The EFI is a self-reported cognitive functioning questionnaire designed for use in community surveys (30). NESARC-III included 12 items from the EFI scales. Nine out of the twelve items fitted a two-factor model, representing two subscales: executive functions (four items) and attention (five items) and a total score, with acceptable reliability and validity (31). Respondents rate each self-statement item in the questionnaire as not at all, a little, somewhat, a lot, or very much. Responses were coded 0-4, with higher values indicating better functioning.

#### Diagnostic Interview for Substance use and DSM-5 Disorders

The diagnostic interview used in NESARC-III was the Alcohol Use Disorder and Associated Disabilities Interview Schedule-5 (AUDADIS-5). It is a structured, computer-assisted diagnostic interview designed for lay interviewers (32). It covers the frequency and amount of drug and alcohol use, DSM-5 substance use disorders and psychiatric disorders.

NESARC-III participants were asked to self-report past year use, including alcohol, tobacco, cannabis, opioids, stimulants, sedatives, club drugs, hallucinogens and others. Participants who reported recreational substance use were asked about the criteria for substance use disorder (SUD).

Consistent with DSM-5, the diagnosis of SUD required at least 2/11 criteria. Recreational substance use that did not meet diagnostic criteria was not included in SUD groups. For test-retest reliability and procedural validity, please see Hasin et al. (32, 33). Although nicotine was found to enhance cognition (34, 35), there is evidence supporting lower cognitive function among smokers compared to non-smokers (35, 36). We also checked our data, and people with tobacco use disorder (TUD) reported lower cognitive function compares to people without SUD. Thus, we decided to include TUD in this study.

The frequency and amount of drug and alcohol use will not be analyzed in this manuscript.

#### Sociodemographic Covariates

Sociodemographic covariates included sex (male, female), race/ethnicity (non-Hispanic Whites, non-Hispanic Blacks, Native, Asian, Hispanic), age (18–90), marital status (married/living with someone as if married, widowed/divorced/separated, never married), education (some high school or less, high school graduate, some college or higher) and past-year household income ($0-$9,999, $10,000-$39,999, $40,000-$109,999, $110,000 or greater).

### Statistical Analysis

Statistical analysis was performed in “R” version 4.1.0 using the “survey” package (37) to implement NESARC-III's complex survey design (Taylor series linearization).

For Table 1, all descriptive results have accounted for survey weights and survey design.

**Table 1:** Demographic characteristics of the sample.

**Variable name**	**No SUD**			**One SUD**			**Two SUDs**			**Three SUDs**			**Four SUDs and above**		
	***N***	**%^**a**^**	**SD^**a**^**	***N***	**%^**a**^**	**SD^**a**^**	***N***	**%^**a**^**	**SD^**a**^**	***N***	**%^**a**^**	**SD^**a**^**	***N***	**%^**a**^**	**SD^**a**^**
**Sex**															
Female	15,307	55.3%	0.50	3,797	45.6%	0.50	878	36.6%	0.48	190	36.7%	0.48	16	29.5%	0.46
Male	10,135	44.7%	0.50	3,890	54.4%	0.50	1,308	63.4%	0.48	284	63.3%	0.48	28	70.5%	0.46
**Race/ethnicity**															
White	12,907	64.3%	0.48	4,545	71.7%	0.45	1,228	68.8%	0.46	240	65.2%	0.48	28	79.9%	0.41
African Americans	5,402	11.7%	0.32	1,586	11.2%	0.32	504	12.5%	0.33	140	18.3%	0.39	13	15.2%	0.36
American Indian/Alaska Native	294	1.3%	0.11	148	2.0%	0.14	48	2.5%	0.16	10	2.9%	0.17	0	0.0%	0.00
Asian/Hawaiian/Other Pacific	1,448	6.6%	0.25	256	3.8%	0.19	64	3.6%	0.19	10	2.3%	0.15	1	2.1%	0.15
Hispanic	5,391	16.1%	0.37	1,152	11.2%	0.32	342	12.5%	0.33	74	11.4%	0.32	2	2.8%	0.17
**Marital status**															
Married/common law	12,633	61.9%	0.49	3,180	52.2%	0.50	694	39.3%	0.49	107	26.5%	0.44	5	16.4%	0.37
Widowed/divorced/ separated	6,486	18.8%	0.39	2,122	21.7%	0.41	546	20.7%	0.41	94	16.3%	0.37	8	22.8%	0.42
Never married	6,323	19.3%	0.39	2,385	26.1%	0.44	946	40.0%	0.49	273	57.2%	0.50	31	60.8%	0.49
**Age (years)**															
18-29	5,020	18.3%	0.39	1,938	25.7%	0.44	816	40.9%	0.49	232	54.1%	0.50	29	63.4%	0.49
30-39	4,551	15.4%	0.36	1,625	19.5%	0.40	536	22.6%	0.42	102	18.3%	0.39	9	20.6%	0.41
40-64	10,833	44.5%	0.50	3,520	46.5%	0.50	786	34.6%	0.48	137	27.1%	0.45	6	16.0%	0.37
65-90	5,038	21.9%	0.41	604	8.3%	0.28	48	1.9%	0.14	3	0.5%	0.07	0	0.0%	0.00
**Education**															
<High school	3,660	12.1%	0.33	1,253	14.8%	0.36	365	15.2%	0.36	87	16.5%	0.37	10	15.8%	0.37
High school/GED	6,539	24.0%	0.43	2,256	28.9%	0.45	707	34.2%	0.47	144	30.8%	0.46	16	44.8%	0.50
Some college or higher	15,243	64.0%	0.48	4,178	56.3%	0.50	1,114	50.7%	0.50	243	52.7%	0.50	18	39.4%	0.49
**Household income**															
$0–$9,999	2,293	6.5%	0.25	864	8.4%	0.28	291	10.5%	0.31	97	16.0%	0.37	12	26.5%	0.45
$10,000–$39,999	10,847	35.0%	0.48	3,611	41.0%	0.49	1,065	43.8%	0.50	252	50.8%	0.50	19	35.5%	0.48
$40,000–$109,999	9,492	42.4%	0.49	2,606	38.8%	0.49	705	36.6%	0.48	106	25.0%	0.43	11	32.5%	0.47
$110,000 or greater	2,810	16.2%	0.37	606	11.9%	0.32	125	9.1%	0.29	19	8.2%	0.27	2	5.5%	0.23
**Past year substance use disorder**															
Alcohol use disorder	–	–	–	2,656	34.6%	0.48	1,874	84.9%	0.36	432	91.3%	0.28	41	88.2%	0.33
Opioid use disorder	–	–	–	88	1.1%	0.10	95	4.8%	0.21	94	19.2%	0.39	33	80.1%	0.40
Tobacco use disorder	–	–	–	4,734	61.8%	0.49	1,913	88.6%	0.32	447	95.3%	0.21	40	93.5%	0.25
Cannabis use disorder	–	–	–	162	2.0%	0.14	368	16.2%	0.37	334	71.3%	0.45	40	92.3%	0.27
Sedative use disorder	–	–	–	25	0.3%	0.05	31	1.6%	0.13	33	6.3%	0.24	24	61.2%	0.49
Stimulant use disorder	–	–	–	11	0.1%	0.03	77	3.2%	0.18	73	15.5%	0.36	34	71.6%	0.46
Hallucinogen use disorder	–	–	–	3	0.0%	0.01	1	0.0%	0.02	0	0.0%	0.00	14	28.4%	0.46
Solvent use disorder	–	–	–	1	0.0%	0.01	2	0.2%	0.04	0	0.0%	0.00	8	20.0%	0.41
Club drug use disorder	–	–	–	0	0.0%	0.00	8	0.4%	0.06	7	0.8%	0.09	20	34.0%	0.48
Other use disorder	–	–	–	7	0.1%	0.03	3	0.2%	0.04	2	0.3%	0.05	1	0.7%	0.08

a *weighted in order to account for the complex survey design of the NESARC-III. SUD, substance use disorder. The weighted percentages are of the entire participants in the group*.

The regression models presented in Tables 2, **4** were estimated using Ordinary Least Squares (OLS) with controls for sociodemographic characteristics (sex, race, marital status, age, education and income). Linearity diagnostic were checked through visual inspection of scatterplots. Estimation was done using the “svyglm” function (with a “gaussian” distribution and an “identity” link function). To test whether sex has a moderation effect on the association between Number of SUDs and cognitive functions, we added an interaction between sex and number of SUDs to the model, while still controlling for the sociodemographic factors. Regression coefficients were transformed into standardized effect sizes equivalent to Cohen's d related to one additional SUD by dividing it by the standard deviation of the model residuals.

**Table 2:** Linear regression estimating the association between the number of substance use disorders and Executive Function Index scores.

**Variable**	**Attention subscale**		**Executive subscale**		**Total score**	
	**Coefficient**	**95%CI**	**Coefficient**	**95%CI**	**Coefficient**	**95%CI**
Number of SUDs—unadjusted	−0.66^***^	[−0.73, −0.59]	−0.45^***^	[−0.52, −0.39]	−1.12^***^	[−1.23, −1.00s]
Number of SUDs—adjusted	−0.64^***^	[−0.72, −0.56]	−0.40^***^	[−0.47, −0.33]	−1.04^***^	[−1.16, −0.92]
*N*	35,916	35,916	35,916			

*CI, confidence interval; SUD, substance use disorder. The results presented in the second row are adjusted for sociodemographic variables. The results were weighted in order to account for the complex survey design of the NESARC-III*.

*** *p < 0.001*.

All results were considered statistically significant if the *p*-value was below 0.05.

## Results

### Sample Characteristics

#### Demographics

Participants with polysubstance SUDs tended to be skewed toward young males, with lower education and household income. Tobacco, alcohol and cannabis were the most prevalent use disorders. Demographic characteristics of the sample are presented in Table 1, weighted means and population shares (percentage) were calculated for continuous and categorical characteristics, respectively. Numbers in Table 1 are reported for five separate groups by the number of Substance Use Disorders (SUDs), from no SUD to four SUDs and above.

#### EFI Scores by Number of SUD and Sex

**Figure 1:**
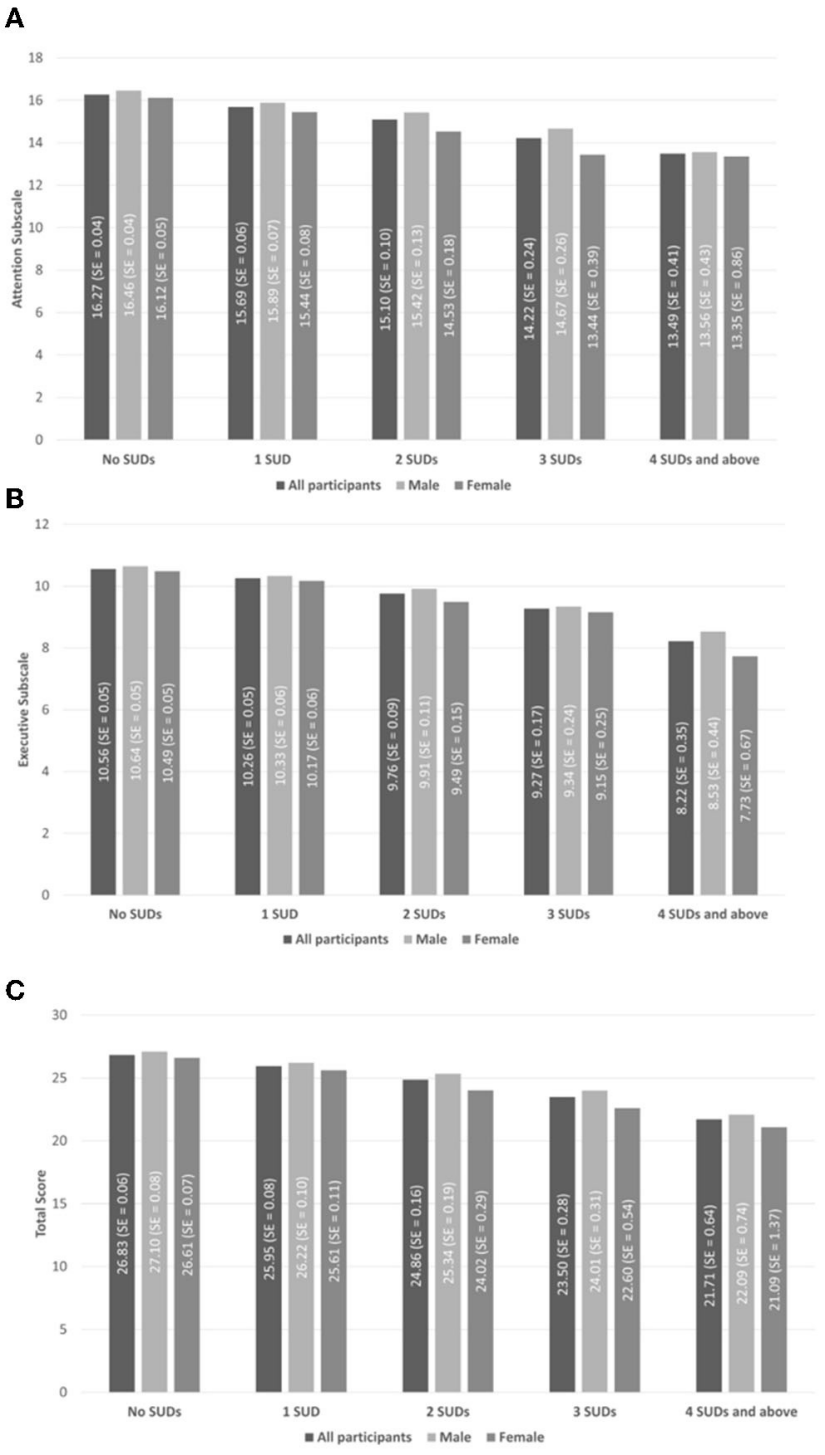
Estimated marginal means of executive function index (EFI) scores and respective standard error (SE) (means and SE adjusted for all above mentioned sociodemographic controls except sex) for males, females, and all participants by number of substance use disorders (SUDs). **(A)** The attention subscale (maximal score = 20). **(B)** The executive function subscale (maximal score = 16). **(C)** The total score (maximal score = 46). Values presented here were adjusted using NESARC-III Survey weights but not for covariates.

Figure 1 presents the estimated marginal means of EFI scores and respective standard error (SE) (means and SE adjusted for all above mentioned sociodemographic controls except sex) for males/females/all participants by the number of SUDs. It is apparent that mean scores decline with additional SUDs. In addition, self-reported cognition seems to be higher for males vs. females.

### Association Between the Number of SUDs and EFI Scores

For our main hypothesis, using three OLS regressions (one for each EFI scale), we test the statistical significance of the association between the number of SUDs and EFI score. A negative and significant coefficient for the “Number of SUDs” measure, mean there is a negative association between number of SUDs and EFI scores. Or in other words, an additional SUD is associated with a decrease in self-reported cognition. Table 2 presents both adjusted and unadjusted results.

The number of SUDs is significantly associated with worse self-reported cognition in all subscales and in the total score, in both the adjusted and unadjusted models. In the adjusted models, on average, an additional SUD is associated with a decrease of 0.64 [95% CI = −0.72 to −0.56], 0.40 [95% CI = −0.47 to −0.33] and 1.04 [95% CI = −1.16 to −0.92] points in the attention, executive function and the total scores, respectively.

### Association Between the Number of SUDs and EFI Scores by Sex

Table 3 presents the prevalence of specific SUD, by sex and by number of SUDs. For our second hypothesis (presented in Table 4), we tested the same association while interacting sex with Number of SUDs. The “Number of SUDs: Females” measure is the association between number of SUDs and EFI score for females only and the same goes for “Number of SUDs: Males.” Table 4 also includes the *p*-value of testing whether the difference between the male and female association is statistically significant.

**Table 3:** The prevalence of specific substance use disorder (SUD), by sex and by number of SUDs.

**Variable name**	**One SUD**	**Two SUDs**	**Three SUDs**	**Four SUDs and above**
	**%^**a**^**	**%^**a**^**	**%^**a**^**	**%^**a**^**
**Male**				
Alcohol use disorder	37.2%	86.2%	93.1%	83.8%
Opioid use disorder	0.9%	3.4%	17.8%	84.7%
Tobacco use disorder	59.3%	88.6%	96.1%	90.8%
Cannabis use disorder	2.4%	17.4%	74.1%	90.2%
Sedative use disorder	0.1%	0.9%	4.0%	64.5%
Stimulant use disorder	0.0%	2.7%	13.9%	73.5%
Hallucinogen use disorder	0.0%	0.0%	0.0%	21.8%
Solvent use disorder	0.0%	0.3%	0.0%	23.9%
Club drug use disorder	0.0%	0.5%	1.0%	33.3%
Other use disorder	0.0%	0.0%	0.0%	1.0%
**Female**				
Alcohol use disorder	31.5%	82.6%	88.2%	98.8%
Opioid use disorder	1.2%	7.2%	21.7%	69.2%
Tobacco use disorder	64.9%	88.7%	93.9%	100.0%
Cannabis use disorder	1.5%	14.0%	66.5%	97.2%
Sedative use disorder	0.5%	2.9%	10.2%	53.4%
Stimulant use disorder	0.2%	4.0%	18.3%	66.9%
Hallucinogen use disorder	0.0%	0.0%	0.0%	44.1%
Solvent use disorder	0.0%	0.0%	0.0%	10.8%
Club drug use disorder	0.0%	0.1%	0.5%	35.9%
Other use disorder	0.2%	0.4%	0.8%	0.0%

a *weighted in order to account for the complex survey design of the NESARC-III. SUD, substance use disorder*.

**Table 4:** Linear regression estimating the association between the number of substance use disorders and executive Function Index scores by sex.

	**Attention subscale**			**Executive subscale**			**Total score**		
	**Coef**.	**Effect size**	**95% CI**	**Coef**.	**Effect size**	**95% CI**	**Coef**.	**Effect size**	**95% CI**
# of SUDs: Females	−0.74^***^	−0.23	[−0.86, −0.63]	−0.44^***^	−0.14	[−0.53, −0.35]	−1.19^***^	−0.24	[−1.36, −1.01]
# of SUDs: Males	−0.56^***^	−0.17	[−0.65, −0.47]	−0.38^***^	−0.12	[−0.46, −0.29]	−0.94^***^	−0.19	[−1.07, −0.81]
*P*-value of Female-Male difference		0.002			0.246			0.008	
*N*		35,916			35,916			35,916	

*CI, confidence interval; SUD, substance use disorder. The results presented are controlled for sociodemographic variables. The results were weighted in order to account for the complex survey design of the NESARC-III. Regression coefficients measure the association between number of SUDs and EFI score, for males and females separately. P-values tests the significance of difference between female and male coefficients and was tested by adding an interaction between number of SUDs and gender to the model. The presented results were derived from the interacted model. Effect size are equivalent to Cohen's d and measure the association related to one additional SUD by dividing it by the standard deviation of the model residuals. P-values tests the significance of difference between female and male coefficients*.

*** *p < 0.001*.

As seen in Table 4, the association between the number of SUDs and self-reported cognition is found to be significant for both females and males. In addition, the interaction between sex and number of SUDs was significant for both the Attention subscale and Total score.

Attention subscale was found to be significantly more associated to number of SUDs for females than for males (*p*-value = 0.002). Among females, an extra SUD condition is related to a decrease of 0.74 [95% CI = −0.86 to −0.63, Cohen's d = −0.23] in the attention subscale, while among males the decrease was found to be smaller at 0.56 [95% CI = −0.65 to −0.47, Cohen's d = −0.17].

In contrast, the executive subscale was found not to be significantly more associated to number of SUDs for females than for males (*p*-value = 0.246). Among females, an extra SUD condition is related to a decrease of 0.44 [95% CI = −0.53 to −0.35, Cohen's d = −0.14] in the executive subscale, while among males the association was found to be −0.38 [95% CI = −0.46 to −0.29, Cohen's d = −0.12].

Total EFI scale was found to be significantly more associated to number of SUDs for females than for males (*p*-value = 0.008). Among females, an extra SUD condition is related to a decrease of 1.19 [95% CI = −1.36 to −1.01, Cohen's d = −0.24] in the total scale, while among males the decrease was found to be smaller at 0.94 [95% CI = −1.07 to −0.81, Cohen's d = −0.19].

## Discussion

The present study provides novel insights on the relationship between polysubstance use and cognitive function. Using data obtained from a large sample of U.S. adults, we sought to determine whether self-reported cognition varied across groups of individuals with differing numbers of SUDs. After controlling for relevant socio-demographic variables, our linear regression models indicate a significant negative association between cognition scores and increasing numbers of SUDs. In both males and females, having more SUD diagnoses was associated with worsening scores in both EFI subscales and total EFI ratings. Our sex-based analysis revealed that the decline in cognitive function observed with the presence of additional SUDs was significantly greater in females.

Previous studies have associated polysubstance use with greater deficits in cognition compared to single drug use (14, 15). It has been proposed that sequential or simultaneous use of multiple drugs may have additive neurotoxic effects, resulting in greater impairment in polysubstance users compared to single drug users (20, 38). In line with these findings, our results could indicate cumulative deleterious effects of additional substances, explaining the decline in EFI scores with increasing numbers of SUD. Alternatively, pre-existing deficits in cognitive function may predispose certain individuals to developing SUDs by more impulsive use of substances (39, 40). Our results may therefore be indicative of premorbid cognitive ability, with higher numbers of SUDs resulting from poorer baseline cognition. Due to our cross-sectional methodology, temporal relationships and consequently the direction of causality between these factors cannot be established. Similar findings of increasing severity with greater numbers of substances have been found for other clinical outcomes including mental distress, psychiatric disorders, and medical issues (3, 41). Longitudinal assessments of cognitive markers in different groups of polysubstance users are required in order to draw further conclusions from our findings.

Our analyses showed a moderating effect of sex on the relationship between polysubstance use and cognition. Higher numbers of SUDs were associated with a significantly greater decline in scores on the attention subscale and total EFI ratings reported by females. Studies investigating sex-differences in polysubstance use are limited; however, current evidence indicates that females may experience more severe cognitive deficits in the context of single-substance use compared to males (28). Neuroimaging studies investigating adolescent alcohol use have shown greater alterations in neural activation patterns as well as smaller prefrontal volumes in females (42, 43). Adolescent females also appear to be more vulnerable to deficits in working memory and executive function associated with cannabis use (27, 28). The faster rate of maturation of prefrontal networks among females may explain their increased susceptibility to cognitive deficits during adolescent onset of substance use (28). In addition, sex differences in the prevalence of psychiatric disorders among substance users have been previously reported and may have contributed to our results (44). Concurrent diagnoses of major depression and posttraumatic stress disorder are more common among females with SUDs compared to males (26, 45). Notably, these diagnoses involve pervasive impairments in cognitive functioning (46, 47), and have been associated with polysubstance use (3). The possibility that differences in psychiatric comorbidities contributed to the sex-specific findings in our sample warrants further investigation.

The results from this study should be considered in light of certain limitations. Our ability to establish temporal relationships between variables and infer causality from our data was limited by the cross-sectional study design. Past-year diagnoses of SUDs were associated with self-assessments of current cognitive function, therefore our results do not account for the timing of SUD onset relative to changes in cognition over time. The cognitive outcomes included in the survey were limited to the Executive Function Index, therefore our conclusions cannot be generalized to other cognitive domains such as learning and memory. Additionally, the clinical outcomes assessed by the NESARC survey were obtained through self-reports and are subject to recall error. The NESARC also surveyed adults aged 18 and over, which limits our ability to generalize our findings to adolescent populations who report high rates of polysubstance use (15). It is also important to note that our definition of substance use was restricted to cases meeting DSM-5 criteria for SUDs and did not include subthreshold drug use. We also did not take frequency or amount of consumption into account, nor did we distinguish between simultaneous and sequential drug use. Whether our findings would vary with other definitions of drug exposure (e.g., misuse, abuse) or according to different patterns of use is unknown. We also did not investigate the contributions of different substances or their combinations, which could have differential effects on the severity of outcomes. For instance, the cognitive deficits associated with chronic alcohol use may be more frequent and severe than those observed in cocaine or cannabis users (48, 49). The effects of heavy alcohol consumption were not addressed in our analysis and should be accounted for in future research. There are additional clinical and lifestyle factors to consider that may have contributed to our findings. In addition to depression and PTSD, co-occurring personality disorders as well as history of childhood trauma are associated with cognitive dysfunction (50, 51). The frequent malnutrition and medical problems seen in this population have also been associated with impairments in cognition (52). Finally, the power of our analyses may have been limited by the small sample size of certain groups when considering individuals with many SUDs. Despite these limitations, we observed a strong dose-dependent relationship between past-year numbers of SUDs and cognition. Our current understanding of SUDs implies that substance use must significantly interfere with an individual's life and functioning (53). Regardless of which drugs are used, higher numbers of SUDs reflect increasing levels of distress and harm, which impair an individual's ability to function. It is likely that the cumulative impact of increasing numbers of SUD diagnoses underlies our findings, rather than the substances themselves.

The current study has important implications in the understanding of SUD patients who often present with extensive polysubstance use and cognitive impairments. Due to its severe and persistent presentation, the treatment of polysubstance use remains a significant challenge in clinical practice. Despite reporting higher levels of psychological distress (41), individuals affected by multiple SUDs are less likely to seek treatment than those suffering from only one (5). Even in clinical settings, these patients are at a heightened risk of treatment dropout, which may result from the severity of their cognitive profile (2). We have provided novel evidence of a sex-specific relationship between cognitive deficits and multiple SUDs. We suggest that these variables be carefully considered when assessing patients and establishing individualized treatment plans. The present study as well as future research will allow the development and implementation of more effective therapeutic approaches, leading to improved outcomes in this understudied population.

## Data Availability Statement

Publicly available datasets were analyzed in this study. This data can be found at: https://www.niaaa.nih.gov/research/nesarc-iii.

## Ethics Statement

The studies involving human participants were reviewed and approved by Centre for Addiction and Mental Health Research Ethics Board. Written informed consent for participation was not required for this study in accordance with the national legislation and the institutional requirements.

## Author Contributions

DR-K, AH, MS, and BL contributed to conception and design of the study. DR-K performed the statistical analysis. ZB and DR-K wrote the first draft of the manuscript. All authors contributed to manuscript revision, read, and approved the submitted version.

## Funding

DR-K was supported by the O'Brien Scholars Program within the Child and Youth Mental Health Collaborative at CAMH and The Hospital for Sick Children, Toronto, Canada. BL is supported by CAMH, a clinician-scientist award from the department of Family and Community Medicine of the University of Toronto and a Chair in Addiction Psychiatry from the department of Psychiatry of University of Toronto.

## Conflict of Interest

BL has obtained funding from Pfizer (GRAND Awards, including salary support) for investigator-initiated projects. BL has participated in a session of a National Advisory Board Meeting (Emerging Trends BUP-XR) for Indivior Canada and has been consultant for Shinogi. He has some in-kind donation of cannabis product from Aurora and medication donation from Pfizer and Bioprojet and was provided a coil for TMS study from Brainsway. He has obtained industry funding from Canopy (through research grants handled by CAMH or University of Toronto), Bioprojet, ACS and Alkermes. He has received in kind donations of nabiximols from GW Pharma for past studies funded by CIHR and NIH. The remaining authors declare that the research was conducted in the absence of any commercial or financial relationships that could be construed as a potential conflict of interest.

## Publisher's Note

All claims expressed in this article are solely those of the authors and do not necessarily represent those of their affiliated organizations, or those of the publisher, the editors and the reviewers. Any product that may be evaluated in this article, or claim that may be made by its manufacturer, is not guaranteed or endorsed by the publisher.
